# Improving the quality of care for people who had a stroke in a low‐/middle‐income country: A qualitative analysis of health‐care professionals’ perspectives

**DOI:** 10.1111/hex.13027

**Published:** 2020-01-22

**Authors:** Leonard Baatiema, Ama de‐Graft Aikins, Fred S. Sarfo, Seye Abimbola, John K. Ganle, Shawn Somerset

**Affiliations:** ^1^ Noguchi Memorial Institute for Medical Research University of Ghana Legon Ghana; ^2^ Institute of Advanced Studies University College London London UK; ^3^ Kwame Nkrumah University of Science & Technology Kumasi Ghana; ^4^ Department of Medicine Komfo Anokye Teaching Hospital Kumasi Ghana; ^5^ School of Public Health University of Sydney Sydney NSW Australia; ^6^ School of Public Health University of Ghana Legon Ghana; ^7^ Faculty of Health University of Canberra Canberra ACT Australia

**Keywords:** evidence‐based care, health policy, low‐/middle‐income countries, quality improvement, quality of care, strategies, stroke, stroke care

## Abstract

**Background and Objective:**

Efforts to improve the adoption of evidence‐based interventions for optimal patient outcomes in low‐/middle‐income countries (LMICs) are persistently hampered by a plethora of barriers. Yet, little is known about strategies to address such barriers to improve quality stroke care. This study seeks to explore health professionals’ views on strategies to improve quality stroke care for people who had a stroke in a LMIC.

**Methods:**

A qualitative interview study design was adopted. A semi‐structured interview guide was used to conduct in‐depth interviews among forty stroke care providers in major referral centres in Ghana. Participants were from nursing, medical, specialist and allied health professional groups. A purposive sample was recruited to share their views on practical strategies to improve quality stroke care in clinical settings. A thematic analysis approach was utilized to inductively analyse the data.

**Results:**

A number of overarching themes of strategies to improve quality stroke care were identified: computerization and digitization of medical practice, allocation of adequate resources, increase the human resource capacity to deliver stroke care, development of clinical guideline/treatment protocols, institutionalization of multidisciplinary care and professional development opportunities. These strategies were however differentially prioritized among different categories of stroke care providers.

**Conclusion:**

Closing the gap between existing knowledge on how to improve quality of stroke care in LMICs has the potential to be successful if unique and context‐specific measures from the views of stroke care providers are considered in developing quality improvement strategies and health systems and policy reforms. However, for optimal outcomes, further research into the effectiveness and feasibility of the proposed strategies by stroke care providers is needed.

## 1 INTRODUCTION

Delivery of high quality and evidence‐based health care is a major imperative worldwide. Although major strides have been made in most countries with regard to developing high‐quality health services towards better patient outcomes over the past two decades, major variations in health‐care delivery still exist and this is quite pervasive in most health‐care facilities in low‐/middle‐income countries (LMICs), where substandard care is provided to patients culminating in poor patient outcomes.^1^, ^2^ For example, it is estimated that up to 8.4 million deaths are recorded annually in LMICs as a result of compromised quality of care.^2^ Within the context of stroke care, a paradoxical situation exists where delivery of quality and evidence‐based care for optimal patient outcomes is relatively limited and poor in LMICs compared to high‐income countries (HICs), although about 80% of the entire global burden of stroke is reported from LMICs.^3^ This burden has been largely attributed to the poor quality of care delivered in those settings and the low uptake of evidence‐based practice.^4^, ^5^, ^6^, ^7^ Findings from the recent INTERSTROKE study further underscore the poor and limited access to optimal stroke care, leading to poor patient outcomes in LMICs.^8^

Similar to other resource‐poor regions, recent research indicates access and delivery of quality and evidence‐based stroke care in Africa is limited and often poor.^3^, ^4^, ^8^, ^9^, ^10^ Studies in Ghana,^5^ Senegal 6 and Congo 7 further exemplify the limited nature of access to quality and evidence‐based stroke care in LMICs. Efforts to improve uptake of better stroke care interventions to ensure optimal patient outcomes in resource‐poor settings have been persistently hampered by a range of barriers such as lack of skilled health personnel, patients’ lack of funds to meet health‐care costs and inadequate health infrastructure.^4^, ^11^, ^12^ In LMICs, health systems are often fragmented and underfunded, and there are rarely organized stroke care services.^13^ Compared to HICs,^14^ quality improvement initiatives for stroke care are rare in LMICs. Efforts to develop interventions and inform relevant policy to improve quality stroke care in LMICs are often obstructed by limited contextualized evidence, a situation occasioned by the fact that most of the studies with insights on improving quality of stroke care are reported from HICs.^15^, ^16^

There is consequently a significant deficit in our understanding of practical and contextually rich insights from stroke care providers on strategies to closing the current widened knowledge‐practice gap in stroke care for improved outcomes in LMICs. To gain better understanding of how to improve the current level of care provided to people who had a stroke, it is critical to gain insights about what it takes to improve the quality of stroke care at the clinical interface. Importantly, being direct care providers for people who had a stroke, and practically involved in almost every aspect of the care continuum within the organizational context of clinical care, perspectives and experiences of health practitioners on practical strategies to improve quality delivery of stroke care are essential. To this end, insights from stroke care providers have the potential to inform relevant policy and quality improvement initiatives for stroke care with more pronounced and sustainable clinical effects.

## 2 MATERIALS AND METHODS

### 2.1 Aim

This study aims to offer insights on clinically relevant strategies to improving quality of stroke care in LMICs through a qualitative analysis of stroke care providers’ recommended strategies, preferences, and expectations to policy makers and health managers regarding quality improvement efforts for stroke care.

### 2.2 Study design

This study forms part of larger qualitative in‐depth semi‐structured interviews conducted over a six‐month period (November 2015‐April 2016) to examine the knowledge‐practice gap in providing evidence‐based care for people who had a stroke in Ghana. The study was conducted in six major referral centres in Ghana. These comprised three tertiary‐teaching and three regional hospitals drawn from the northern, middle and southern belts of Ghana. These sites were purposively selected to account for the different geographical and socio‐economic contexts across Ghana. Where applicable, the study followed the Consolidated Criteria for Reporting Qualitative Research (COREQ) checklist for reporting qualitative research.^17^

### 2.3 Participants

Forty acute stroke care professionals, comprising nursing, medical, physician specialists and allied health, were recruited into the study (see Table 1). Participants were drawn from these various professional backgrounds and clinical settings to ensure rich and diversified insights. They were each chosen because they play a key role in delivering stroke care and have made decisions or played supervisory roles in relation to the delivery of stroke care.

**Table 1:** Demographic information of study participants (N = 40)

Number	N	Percentage (%)
Sex		
Male	17	42.5
Female	23	57.5
Age		
25‐35	7	17.5
36‐45	21	52.5
46‐55	9	22.5
56+	3	7.5
Years of professional experience		
2‐5	8	20
6‐9	17	42.5
10‐15	12	30
15‐20	3	7.5
Professional background		
Nurse	20	50
Neurologist consultant	1	2.5
Neurologist	1	2.5
Emergency physician specialist	1	2.5
Medical officer/physician	9	22.5
Clinical psychologist	2	5
Physiotherapist	5	12.5
Dietitian	1	2.5

### 2.4 Recruitment and sampling

Participant recruitment was conducted based on a standard protocol for recruiting participants for research in Ghanaian hospital settings. Formal invitation letters, ethics clearance letters and study information sheets were sent to all study sites for approval either by the hospital administrators, heads of units and departments as appropriate prior to actual recruitment and conduct of the interviews. The study employed a mixed sampling approach. First, a maximum variation principle guided the sampling process to ensure recruited participants had diverse clinical experiences, sex, age group, years of working experience, different professional ranks and covering a range of schedules/roles in the stroke care continuum. Second, with the help of the hospital administrators or unit heads, a purposive sampling technique was utilized to recruit health‐care professionals who were directly involved or had expertize in the provision of acute stroke care. Prospective study participants were briefed about the study, and interviews were then scheduled for those who consented to participate in the study. Interviews were conducted until theoretical data saturation was reached, a point where no new information emerged from subsequent interviews.

### 2.5 Data collection

Semi‐structured, face‐to‐face in‐depth interviews were conducted in English by the first author. Interviewing took place in various settings including consulting rooms, hospital wards and conference rooms. An interview guide was designed and used to facilitate the conduct of interviews. The interview guide was open‐ended in nature with follow‐up prompts to seek clarity or gather further insights from participants. The interview guide was designed based on review of relevant literature. Also, a number of stakeholders including carers of people living with a stroke and stroke care experts were consulted and their views incorporated in developing the content of the interview guide. The interview guide was later pre‐tested prior to the data collection and still further refined iteratively during the data collection process to ensure questions were appropriately framed, robust and had the flow to elicit adequate and appropriate responses. The interview guide principally solicited the views of stroke care professionals on practical and innovative strategies to improve quality of acute stroke care.

Participants were not known to the researchers prior to the study though it is likely some may be familiar with the first and second authors given their previous work in some of the study sites. Overall, each interview lasted about 50 minutes. Interviews were audio‐recorded alongside handwritten field notes.

### 2.6 Data analysis

All interviews were audio‐recorded, transcribed verbatim by professional transcribers and anonymized. A thematic content analysis was consequently undertaken,^18^ using an inductive analytical approach where themes were generated based on recurrence and not on a prior framework. The first author (LB) read and re‐read all transcripts on a line‐by‐line basis to establish familiarity with the content of the interviews based on the grounded theory principle.^19^ Five transcripts were first coded, and a coding framework was developed and subsequently applied to all transcripts after reading several transcripts to ensure transparency and systematic coding of the interview transcripts. The coding process was aided by the NVIVO 10.0 (QSR International) program software. The framework was revised iteratively to ensure all relevant information was reported. All codes across the interviews were consolidated into key thematic categories related to strategies to improve acute stroke care. Patterns of codes from different participants were compared to establish similarities and differences. A second author (AdGA) cross‐coded a sample of transcripts independently to establish reliability. A draft of the resulting categories according to the key themes and interpretations was reviewed by two other authors (AdGA and SS). Interview quotes from participants were used to illustrate or support key study findings and to highlight some underlying themes of participant perspectives.

## 3 RESULTS

### 3.1 Basic participants’ characteristics

Overall, a total of forty^20^ participants were interviewed, and their basic characteristics are shown in Table 1 below.

### 3.2 Strategies for improving quality of stroke care

**Figure 1:**
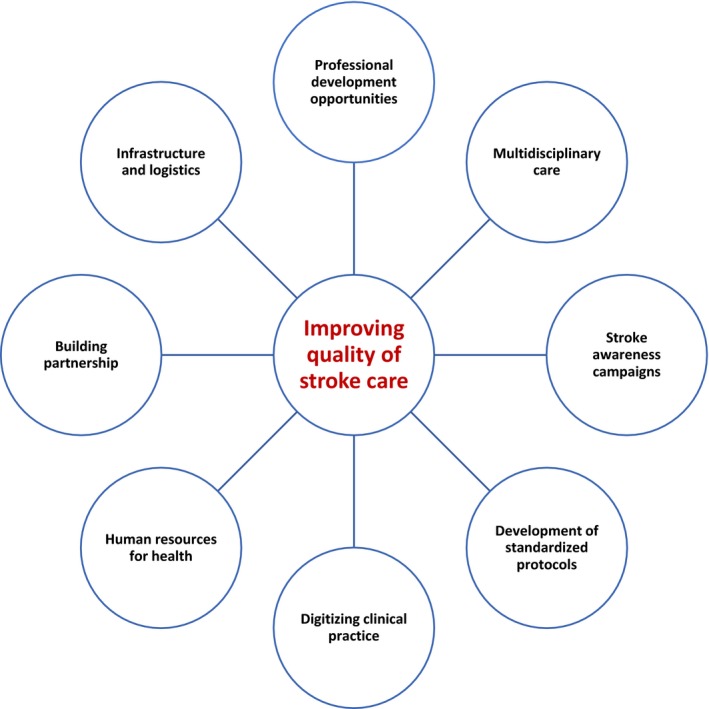
Framework of strategies for improving in‐patient stroke care in LMICs

Participants proffered a wide range of strategies to support efforts towards optimization of acute stroke care in the study settings. Seven overarching thematic strategies emerged from participant narratives (see Figure 1).

### 3.3 Adequate allocation of medical logistics and infrastructure

This theme was consistently emphasized by stroke care professionals. Participants attributed the inconsistency, variations and poor nature of care to limited or an apparent lack of medical logistics and consumables. For instance, essential equipment such as blood pressure monitors and electrocardiograph machines were either not available, of poor quality, or worn out. A participant bemoaned the state of limited medical equipment which often compromise delivery of quality stroke care.

Sometimes when the emergency ward is full, you can come here and find a suspected person with a stroke sitting on the wheel chair, there is no place, or a people who had a stroke comes in and urgently requires oxygen, but other people are on oxygen and you cannot say that let me take patient A oxygen and give to patient B, so in order to meet recommended treatment guidelines to ensure optimal clinical outcomes, there is the need to ensure adequate supply of resources to ensure quality services to patient care. (Nurse, Participant 11).

Some participants (nurses) noted that the inadequacy or complete absence of such resources inevitably affected fidelity to standard treatment practice and procedures. Frequent stock‐outs of essential medical consumables were reported to lead to patients being required to purchase such consumables on their own, which sometimes results in pronounced delays in treatment or increased length of stay. Limited or lack of patient monitoring for bedsores was reported to increase risk of pressure injuries or sores, thus affecting the quality of care provided, overall. Participants suggested that such health system deficiencies and shortages could be addressed with increased political commitment to health care. Some suggested the private sector and philanthropists could play a key role in augmenting the efforts of government to ensure adequate supply of medical resources.

### 3.4 Human resources

Human resources, both in terms of numbers and quality of staff, was discussed and recommended extensively by participants. However, there was some divergence in views, with most of the medical doctors (physicians) tending to suggest high‐quality human resource capacity, whereas others focussed more on staff numbers. They believed access to adequate and sufficiently motivated staff is more critical since to them the training to improve their competence and skills could later be tackled. This according to them could take the form of better remunerational packages, reward systems and scholarship opportunities for career development in stroke care Most of the participants also expressed enthusiasm in providing quality stroke care but this according to them was often undermined by the limited number of staff available resulting in increased workload, stress and fatigue. In terms of how to improve the current acute shortages in health workforce, participants proposed both the private and public sectors could play a role, especially in recruiting and training more health staff in stroke care. The introduction of sub‐specialties in stroke care in health training institutions may also help address the problem. All these could be achieved through increased government allocation for the recruitment and training of health‐care professionals on stroke care as poignantly expressed by one participant in the study.

Increased in staff supply will help address the deficit in human resource capacity for stroke care especially specialists with the requisite skills to provide or supervise the delivery of care to people who had a stroke. This will help reduce the current workloads and ensure staff have ample space and expertise to deliver adequate and appropriate care (Medical Officer, Respondent 3)

Medical doctors mostly from the middle and northern belts of the study sites expressed critical views about the inequity and unfairness in the distribution of health‐care providers in Ghana. Participants suggested the need for equitable distribution since they believe most health staff were concentrated in the urban cities of Ghana. They opined government needed to adopt pragmatic measures to tackle this issue through a quota system to ensure every health facility has the requisite specialist staff to provide optimal and quality stroke care.

Our human resource problem in Ghana is a problem of inequitable distribution of healthcare providers. It is as simple as that and yet there is lack of political will to fix this problem. (Medical Officer, Respondent 2)

### 3.5 Development of standardized protocols

The need for protocols, flow charts or stroke treatment algorithms to guide the continuum of care for people who had a stroke was suggested by participants with three forms of standardized protocols emerging. A protocol or clinical guideline for treatment and management of diagnosed stroke was a predominant theme suggested to improve care. Accordingly, clinical guidelines and protocols for stroke care were reported to be essentially non‐existent and this constrains efforts to provide quality of care and as well as undercutting efforts to adhere to evidence‐based treatment guidelines, resulting in variations and inconsistencies in the delivery of care.

Without treatment guidelines, there is no or little direction and consistency in the care we provide, especially when there is no specialist or senior colleague to guide the care we provide… (Nurse, Respondent 17)

The second was a protocol to guide referral of people who had a stroke. According to participants, this is needed to ensure patients are referred to the appropriate point of care or health facility at the onset of stroke:

Because there are no existing referral protocols, the patient will go to a clinic, the clinic will admit them before later making referral. Even though the patient attended a health facility, was that health facility the right place for the person to have gone? They went there, and they could not handle it and they are now bringing it, and most of the times these clinics will call to notify about a referral case only when the conditions are deteriorating and because they don’t want mortalities recorded in their facilities, they will just call in to inform us they are referring a patient to our facility (Nurse, Respondent 7)

Medication dispensary protocol was also highly recommended by nurses given the considerable delays they face at the hospital pharmacies or drug dispensary points. Family members join long queues to purchase medications following prescription by a medical doctor. Since most hospitals adopt a “first come, first serve policy”, they often overlook family members with patients in critical conditions such as a stroke, and delays in accessing drugs potentially worsen the health outcomes of patients.

You go there, and you see a long queue, and everybody is in a hurry, nobody knows which one is an emergency case. At least if there was an existing protocol or policy to ensure particular patients require immediate attention should always be prioritized and served upon arrival. Better still, I think provisions should be made to ensure mini dispensary points are located in every ward (Nurse, Respondent 31)

### 3.6 Computerization and digitization of medical practice

With difficulties in tracing patient records, medical history and delays in transferring records from one unit/division of care to the other, majority of the participants recommended the need to computerize all relevant medical records. In their view, this has the potential to minimize significant delays in the treatment of acute stroke care where time is critical in the continuum of care. Some care providers, mostly nurses, believed this would facilitate speedy review of medical histories and subsequently support appropriate design of care and discharge plans. They also indicated that adequate capacity building and training workshops to enable them use technology to advance their clinical practice should be prioritized.

Electronic medical records should be introduced to ensure speedy access to accurate patient records. I am aware of other contexts in Ghana where clinical practice has been digitised to make it possible for patients’ information to be shared between departments or units among health professionals within seconds. This should be standardised in all clinical settings in Ghana in order to minimise delays in clinical decision making and potentially longer waiting times for patients will be minimised (Medical Officer, Respondent 22)

A few participants however expressed reservations about the underdeveloped nature of existing medical technology and Internet connectivity services. Accordingly, this has the tendency to delay delivery of care in the event of a total blackout or Internet connectivity problems. Some were apprehensive, noting that any loss of patient electronic information without any form of backup will have dire consequences for patient care.

Yes, but you know applying technology in medical practice is yet to be developed in this part of the world despite considerable boom in technological advancement globally. Internet connection and power supply is erratic in this part of the world, and this doesn’t support digitization of medical practice in this part of the world (Physiotherapist, Respondent 15)

### 3.7 Stroke awareness campaigns

Most participants interviewed agreed there was a need for public health campaigns to create awareness on early symptoms and signs of a stroke which was observed to be quite poor. They intimated that the majority of Ghanaians are not able to identify early stroke signs and are unsure about immediate steps to take following early signs and symptoms of a suspected stroke. This according to them could be a plausible cause of patient delays in seeking care following stroke onset with most presenting late at emergency departments.

Public awareness on early stroke signs and symptoms is very poor. Most of the patients and their relatives who came in here were unaware it was a stroke, so public awareness and education programmes for the public to know some early stroke signs and symptoms will be helpful. (Medical Officer, Respondent 5)

Despite consensus among participants on the need to intensify public awareness on early stroke signs and the need to seek medical attention, participants were divided as to the form or strategy this campaign should take. Some recommended channels such as television and radio, and other participants were of the view that stroke awareness campaigns should take the form of community‐based activities such as durbars, plays, or using outreach programmes. Others suggested a combination of social media, traditional media and use of community durbars to create such awareness at the population level.

On the suggestion of using community‐based durbars and drama, one participant noted:

Due to low literacy among many rural community members, awareness creation on early stroke signs should rather take the form of contextually‐relevant campaigns in their own settings, language, and vocabulary. This will have more impact than rolling out a one‐size‐fits all media campaign… (Nurse, Respondent 35)

Although not a widely expressed view, a cross‐section of participants opined a key point in relation to rolling out public education events on early stroke signs and symptoms. Accordingly, public awareness campaigns on the early signs and symptoms of stroke could be mainstreamed into the health‐care system where health‐care professionals are required to undertake targeted public education on stroke during consultancy, in‐patient care or out‐patient visits for people with high risk of stroke. This was strongly emphasized by participants as a short‐term measure in light of the lack of government support for public education for stroke.

### 3.8 Institutionalize multidisciplinary care team

A key feature of evidence‐based stroke care is the delivery of care by a multidisciplinary team care. Unsurprising, a consensus emerged among participants especially the allied health staff about the need to institutionalize this approach, which is currently lacking in how care is delivered to people who had a stroke. As a result, most of the allied health professionals suggested a need to ensure adequate measures to ensure care is provided by a multidisciplinary team. Participants emphasized the need for care plans, especially discharge care plans, to be developed by a multidisciplinary team. The commentary below encapsulates a view underscoring why delivery of care to people who had a stroke should be multidisciplinary:

The challenge that still remains is the fact that a patient could even be discharged before your general rounds, ……… so you go there and maybe there was a patient you attended to the previous day and when you get there the patient is discharged so you missed out on that patient. It is possible the patient will get home after discharge and continue on a diet which may not inure to her/his benefits, and in no time, the patient is on admission again with a similar or exacerbated condition (Dietitian, Respondent 12)

Some participants were of the view that though such teams existed in their hospitals, they are not operationalized, do not hold regular meetings and rarely provide care as a multidisciplinary team. Regular multidisciplinary team meetings were emphasized for facilities where such teams existed and were functional. The accounts of a participant summarized this point:

I think it is important we meet as a team from the various units. Such team meetings should not be considered as a fault‐finding meeting as this may discourage attendance. It should be a platform for all to attend, bring out the problems they face in providing care and solutions sought to improve patient care. (Nurse, Respondent 29)

In line with this, one participant recommended the need to exploit available technological tools by creating e‐platforms such as WhatsApp to promote collaborative or multidisciplinary care.

The physiotherapy department has shared with our unit in charge (head) a WhatsApp number so when a person with a suspected stroke is admitted into our ward or any patient requiring the services of a physiotherapist, we just WhatsApp them, provide the name of the ward, the patient bed number, his/her name and then they come promptly to attend to the patient. This should be encouraged and extended to other units within the hospital (Physiotherapist, Respondent 19)

### 3.9 Professional development and training opportunities

Emphasizing limited knowledge and competence in providing quality care for people who had a stroke, most participants recommended uninhibited access to professional development and training programmes for health professionals who provide care for stroke and other NCDs. They regard this as a career development incentive and acknowledged that although professional training programmes are sometimes organized, they often lack financial support to participate in such training workshops. A recommendation was made for budgetary allocation to sponsor health‐care providers. Opportunities for relevant conference and workshop attendance would ensure stroke care providers are current with the latest clinical guidelines.

The sponsorship bit is essential because most of the refresher training workshops or continuing education in any professional course or field is expensive in recent times and so we will need financial support from government and hospital management to develop our profession further (Nurse, Respondent 40)

Another had this to say:

I need it, because sometimes when you are on duty, and some stroke cases arrive, then I realised I've forgotten some basic procedures, because I left the classroom for a very long time (Nurse, Respondent 16)

Others were of the view that opportunities for refresher and professional development workshops would ensure currency with contemporary clinical guidelines for stroke and other chronic conditions. In their views, such opportunities would enable them to learn more about current best practices and new strategies to achieving best clinical outcomes for patients. Related to this is internal staff education and training, which could be arranged during monthly departmental meetings. This was viewed as a cost‐effective way to improve knowledge and skills in quality stroke care delivery.

### 3.10 Building partnerships for stroke care improvement

The analysis also found that building partnership across disciplines, within and outside hospitals, including outside the country, featured prominently as a key strategy to improve the quality of stroke care. Participants, mostly medical doctors, indicated a need for care improvement partnerships or collaborations to make it possible to train staff on the provision of stroke care. In their view, such partnerships should be supported by the state, especially senior health managers and policy makers by actively exploring and sponsoring such partnerships. These partnerships should support specialists from other well‐developed health systems to visit and share skills, experiences and knowledge on stroke treatment and management.

I think government and our policy makers should begin to prioritise support for partnership building between hospitals and health professionals to improve care. This is definitely a cost‐effective measure to improve care and safe lives. Specialist in teaching hospitals with well‐established stroke care systems should be able to train staff from health facilities with less developed stroke care systems. Exchange learning visits should be made possible as this could make a significant difference in saving or improving lives of people who had a stroke (Medical Officer, Respondent 8)

Other participants stressed the need for partnerships across countries especially linking countries with established stroke care systems and those with less developed systems of care.

The opportunity to host stroke care specialists from well‐developed stroke care systems of the developed world or even support local staff to travel there to understudy how stroke care is provided will offer fresh perspectives to staff, offer new skills, information and ideas on how to manage stroke will be worthwhile (Medical Officer, Respondent 18)

In summary, a diversity of views from different stroke care professional was expressed in relation to providing optimal patient care. Whilst some views were associated with specific categories of stroke care professionals, most of the recommended strategies were held by most or all participants to be essential.

## 4 DISCUSSION

To our knowledge, the present study is about the first to report the views and perspectives of stroke care professionals on strategies to improve the quality of acute stroke care in low‐/middle‐income clinical settings. Overall, stroke care professionals recommended seven contextually and clinically practical strategies to improve quality of acute stroke care. The findings also highlighted a diversity of perspectives on strategies to improving quality stroke care, reflecting the broad range of health‐care professionals implicated in optimal care. Among the strategies underscored by participants are the need for provision of adequate medical infrastructure and logistics, computerizing clinical practice, development of standardized clinical guidelines to direct delivery of care, promote multidisciplinary team care. The rest relate to support for staff professional development, public awareness campaigns on early signs and symptoms of stroke, partnership‐building to improve stroke care.

Overall, the proffered strategies respond to recent recommendations by the Lancet Neurology Commission on the need to have locally based measures to effectively tackle the growing burden of stroke in LMICs.^21^ These findings largely corroborate previous scholarship on increasing the quality of care in other contexts. First, knowledge exchange and partnerships between low‐/middle‐income countries and high‐income countries has emerged as a key global health strategy to build resources for health crises currently experienced in most countries with underdeveloped health systems.^22^, ^23^, ^24^ Such collaborations are recommended as a strategy to promote the development of clinical skills especially among workers in less developed health‐care systems from LMICs. The current sustainable development goals have placed further importance on this as a measure to build capacity in global health. Thus, the finding in relation to fostering partnerships and collaborations to improve stroke care corroborates international best practice measures to improve care, especially in LIMCs where health‐care capacity is often weak and limited in scope and quality. Within the context of stroke care, there is evidence of a previous partnership between a team from the UK and their Ghanaian counterparts (Wessex‐Ghana Stroke Partnership) which sought to build capacity of stroke care professional in stroke care.^25^ This partnership has since led to stroke care professionals benefiting from professional capacity building opportunities on stroke care (eg, managing continence, discharge planning, improving mood, functional independence and swallowing) and overall improvement in the health‐care systems for stroke care in the beneficiary health facilities. Evidence from South Africa also suggests a similar arrangement appears to show promise.^26^ A review also corroborates the present findings on the need to establish partnerships to improve care. The review highlights that beneficiaries of such exchange or partnership programmes benefit both personally and professionally where skills from such collaborations are often applied to improve patients outcomes.^27^

Participants also emphasized the need for competency‐based training in acute stroke care through professional development and training opportunities, particularly in areas such as diagnostic algorithms for stroke, including the use of stroke scales, and other treatment protocols. Studies in HICs underscore this as part of measures to improve stroke care. For instance, the USA has prioritized training on stroke care for staff in delivering evidence‐based care for optimal patient outcomes.^14^, ^28^ Besides stroke care, advocacy for competency‐based refresher training for staff have increasingly been recognized as an essential tenet of efforts to strengthen the health‐care system and improve the quality of care provided to patients in LMICs.^29^ Hence, the suggestion by participants for professional development and competency‐based training workshops is an imperative.

A key strategy to improving quality of stroke care is ensuring adequate resources to support the delivery of care. Access to medical logistics and infrastructure in LMICs is often limited, and this has been previously recognized to be a persistent issue.^11^, ^12^, ^30^ The findings from this study aligns with these previous studies on the need to ensure adequate provision of medical consumables and infrastructure such as neurosurgical services, neuroimaging and laboratory services to support stroke care. Previous literature shows that human resources for health crisis has persisted in LMICs over the past decades despite modest gains made to tackle this through expanded competency‐based training and mentorship schemes.^30^, ^31^, ^32^ Despite this, the problem persists and has been highlighted as a major impediment to efforts to reduce the growing burden of NCDs such as stroke.^33^ The views expressed by participants about the limited health workforce to provide optimal stroke care accentuate the centrality and pervasiveness of this issue. This revelation by participants needs to provoke interest within key stakeholders in finding sustainable solutions to the problem. Recommendations by participants on the need for adequate human workforce to support stroke care are consistent with similar recommendations in the past.^30^, ^34^, ^35^ These are issues which have also been noted in previous studies conducted in HICs,^36^ suggesting the global dimension of the problem.

Internationally, experts, consensus statements and stroke care literature have consistently emphasized the need to have a multidisciplinary approach to standardize health care.^13^, ^14^, ^20^, ^37^, ^38^, ^39^ Participants in the present study also expressed similar need. As noted by participants, e‐platforms such as WhatsApp can serve as an excellent channel to share information between stroke care professionals. This arrangement could potentially facilitate development of joint care plans for the patients, fosters better and effective communication. Evidence suggests the adoption of multidisciplinary team care for people who had a stroke is still limited in LMICs,^4^, ^11^ notwithstanding some sporadic evidence.^40^

Medical or clinical practice is increasingly becoming digitized following modern technological advancements. As a result, patient clinical information can be stored electronically and retrieved through the same means to support faster clinical decision‐making. However, this revolution is more relevant to HICs where health‐care systems are well‐developed with state‐of‐the‐art medical infrastructure to support digitization.^20^, ^37^ In LMICS, this is increasingly encouraged by stroke care researchers 38, 39, 41 though recent evidence shows uptake is still quite limited.^20^ A study from Australia shows digitizing patient clinical data can improve coordination of care and consequently enhances patient outcomes.^42^

Consistent with the findings from this study, the need for stroke awareness campaigns in LMICs is increasingly being advocated by recent studies.^43^, ^44^ Even among health‐care providers in Africa, identifying early stroke symptoms can sometimes be a challenge at the population level.^45^ Improving the public's knowledge of stroke risk factors, signs and symptoms of stroke is critical to improving the quality of stroke care. Without organized, coordinated and contextualized approaches to educate the public, the full potential of proven therapies for prevention or acute intervention will not be realized.

### 4.1 Strengths and limitations

The recruitment of participants in this study targeted only those with key primary responsibility for stroke care, which may have biased the study towards a particular category of participants. However, the observed divergent views on strategies to improve care across the different category of stroke care professionals imply a diverse set of perspectives on the issue. Thus, the inclusion of different stakeholders in the continuum of stroke care is considered a strength of the study. Some study limitations were also observed. Despite the inclusivity of the study participants, the views expressed by participants may not be representative of the full range of views of all stroke care providers and thus should be considered in interpreting the study findings.

## 5 CONCLUSION

In sum, despite growing interest to close the gap between existing knowledge on how to improve quality of stroke care and standard clinical practice, LMICs still lag behind HICs in providing quality and evidence‐based stroke for optimal clinical outcomes. The present study revealed potential strategies to improve quality in stroke care delivery based on views from stroke care providers. These findings are relevant to inform and shape policy initiatives to improve quality of stroke care in LMICs. In light of a global search for practical and contextual initiatives to improve uptake of evidence‐based stroke care interventions, the recommended strategies to improve stroke care are timely. It is important for policy makers and health managers to consider the recommended strategies in context vis‐à‐vis the different stroke care providers in developing quality improvement initiatives. Any contrary action could potentially hinder or undermine processes to improve stroke care. To ensure prudent allocation and use of the limited resources for health in LMICs, future research needs to evaluate the effectiveness and feasibility of each of the recommended strategies. Identifying the views of people who had a stroke and carers regarding strategies to improve quality stroke care should also be prioritized in future studies.

## CONFLICT OF INTEREST

The authors declare that they have no competing interests.

## AUTHORS' CONTRIBUTIONS

LB, SS and AdGA conceived and designed the study. AdGA. LB, JKG, SA and SSF analysed and interpreted the data. LB drafted the manuscript and later revised after critical feedback from SS, AdGA, SSF, SA, JKG. All authors have reviewed and approved the final manuscript for submission.

## ETHICS

The study protocol obtained and maintained ethical clearance from four institutional review boards (IRB). These comprised the University Human Research Ethics Committee (2015‐154H) and the Ghana Health Service Ethical Review Committee on Research Involving Human Subjects (GHS‐ERC: 11/07/15). Approval was also sought from the Committee on Human Research Publications and Ethics of the School of Medical Sciences of the Kwame Nkrumah University of Science and Technology and the Komfo Anokye Teaching Hospital (CHRPE/AP/141/16) and the Institutional Review Board of the 37 Military Hospital (37MH‐IRB IPN 035/2015). Written consent was obtained from all participants who consented to be interviewed. Written consent was obtained from all participants prior to each interview. Participants were not incentivized or provided with motivational packages for participation. With consent from participants, all interviews were audio‐recorded.

## Data Availability

The data that support the findings of this study are available on request from the corresponding author. The data are not publicly available due to privacy or ethical restrictions.
